# Revisiting the Evidence Underlying Dietary Recommendations on Saturated Fats and Cardiovascular Disease: A Narrative Synthesis of Systematic Reviews and Meta-Analyses

**DOI:** 10.1016/j.advnut.2026.100746

**Published:** 2026-10-07

**Authors:** Julie Marvel Mansfeldt, Martina Abodi, James Thomas Brenna, José M Ordovás, Janet C King, Arne Astrup, Renata Micha, Nina Teicholz, Robert H Eckel, James O Hill, Ronald M Krauss, Carlo Agostoni, Faidon Magkos

**Affiliations:** 1 Department of Nutrition, Exercise and Sports, University of Copenhagen, Copenhagen, Denmark; 2 Department of Clinical Sciences and Community Health, Dipartimento di Eccellenza 2023-2027, University of Milan, Milan, Italy; 3 Pediatric Unit, IRCCS Ca’ Granda Ospedale Maggiore Policlinico, Milan, Italy; 4 Dell Pediatric Research Institute, Dell Medical School, University of Texas Austin, TX, United States; 5 Department of Nutrition and Genomics, Human Nutrition Research Center of Aging, Tufts University, Boston, MA, United States; 6 Department of Nutritional Sciences and Toxicology, University of California-Berkeley, Berkeley, CA, United States; 7 Clinical Nutrition Unit, Copenhagen University Hospital – Bispebjerg and Frederiksberg, Copenhagen, Denmark; 8 Food is Medicine Institute, Friedman School of Nutrition Science & Policy, Tufts University, Boston, MA, United States; 9 Department of Food Science and Nutrition, University of Thessaly, Volos, Greece; 10 Independent Researcher, New York, NY, United States; 11 Department of Medicine, University of Colorado Anschutz Medical Campus, Aurora, CO, United States; 12 Department of Nutrition Sciences, University of Alabama at Birmingham, Birmingham, AL, United States; 13 Departments of Medicine and Pediatrics, University of California-San Francisco, San Francisco, CA, United States

**Keywords:** CVD risk, mortality, SFAs, palmitic acid, diet

## Abstract

The recommendation to limit intake of saturated fatty acids (SFA) remains part of dietary guidelines issued by numerous national and international organizations. We performed a search of previous systematic reviews and meta-analyses on the relationship between SFA intake and cardiovascular disease (CVD) incidence and mortality, including coronary artery disease (CAD) and stroke, and total mortality. We screened 3285 records and included 31 publications [32 systematic reviews/meta-analyses: 23 observational and 9 randomized controlled trial (RCT) based] published between 2001 and 2025. The duration of primary studies ranged from 0.4 to 32 y. The number of assessments included 34 for disease incidence, 39 for mortality, and 2 for composite cardiac endpoints. Most systematic reviews and meta-analyses found no significant relationship between intake of SFA and CVD outcomes. Two found a harmful effect (1 each for CAD and peripheral artery disease), whereas 3 found a beneficial effect (all on stroke in East Asian cohorts). Several others showed mixed results: 6 observational modeling studies suggested harm with SFA compared with polyunsaturated fatty acids (PUFA) for CVD incidence or mortality, whereas 2 RCT-based reviews found increased CVD incidence but no effect on mortality. We conclude that the currently available evidence does not demonstrate a consistent association between dietary SFA intake and cardiovascular events or mortality. Replacing SFA with PUFA appears to be associated with favorable cardiovascular outcomes in observational and well-conducted short-duration clinical research studies. Overall, these findings suggest that recommendations on SFA intake may be more effective when considered within the broader context of dietary patterns rather than replacement of single nutrients.

**Statement of significance**

By integrating evidence from systematic reviews and meta-analyses spanning over 2 decades, this work shows that associations between saturated fat intake and cardiovascular outcomes are inconsistent and largely neutral. The findings emphasize the role of replacement nutrients and dietary patterns, offering a broader framework to inform future dietary guidance.

## Introduction

Cardiovascular disease (CVD), such as coronary artery disease (CAD) and stroke, remains a leading cause of death, accounting for >2 in 10 deaths globally [1]. In the United States, ≥80 million people are living with some form of CVD or have experienced a cardiovascular event, including CAD/myocardial infarction, stroke, or peripheral artery disease [2]. These diseases and their downstream sequelae are costly, amounting to roughly $200 billion in direct costs associated with medical visits and another $100 billion in indirect costs associated with loss of productivity, illness, and death [2].

Poor-quality diets are estimated to be responsible for roughly two-thirds of CAD-related deaths worldwide [3]. This underscores the importance of whole dietary patterns, beyond individual foods or nutrients, for optimal cardiovascular health, a concept that has been variably adopted by contemporary nutrition recommendations around the world [4]. Still, the guidance to specifically limit intake of saturated fat, or SFA, has been a consistent feature of dietary guidelines issued by multiple national and international organizations and health authorities worldwide, dating back to the earliest public nutrition policies [5]. For example, the 2025–2030 Dietary Guidelines for Americans confirmed the recommended limitation of SFA to <10% of total daily calories [6] (https://cdn.realfood.gov/DGA.pdf), the United Kingdom dietary guidelines set this threshold at 11% [7], the American Heart Association at 6% [7], and the European Food Safety Authority suggests intake of SFA should be “as low as possible” [8].

However, this guideline has not been without controversy. For example, given the long time-to-event for several cardiovascular endpoints, most evidence is derived from observational studies, and the randomized controlled trials (RCTs) that have been conducted have generally not been adequately powered [9]. These studies complicate attempts to draw causal inferences despite the large body of data. Furthermore, a growing body of evidence suggests that the health effects of SFA consumption may depend on the food matrix (the physical structure of food that binds nutrients and nonnutrient components together, which influences their physiological behavior and functionality) [10] and the fatty acid chain length (i.e., the number of carbon atoms) [11], [12]. Concern has also been raised that the recommendation for a reduction in SFA could potentially lead to replacement with refined carbohydrates, which themselves have unfavorable health consequences [13], [14]. Some detractors of the guidelines have raised concerns about deficits in the quality of the review process itself [15], [16].

With these nuances in mind, here, we systematically gathered and appraised the currently available literature regarding dietary SFA intake and CVD incidence and mortality.

## Methods

We summarized evidence from previously published meta-analyses and systematic reviews on the consumption of SFA and CVD incidence and mortality, including both CAD and stroke. Systematic reviews and meta-analyses reporting on all-cause mortality were also considered. The review was conducted in accordance with the PRISMA statement [17].

### Eligibility criteria

We included previously published meta-analyses and systematic reviews of RCTs, observational studies (cross-sectional, cohort, case-control), or any combination thereof. We excluded individual primary studies or reanalyses of those studies from our analysis. We specifically focused on hard CVD end points (e.g., CAD/myocardial infarction, stroke) rather than risk factors (e.g., LDL cholesterol, blood pressure, levels of glycemia, and insulin sensitivity). Reviews of both primary and secondary prevention studies were eligible. We excluded any reviews focusing specifically on pregnant women or children.

There were no restrictions on publication date, but only systematic reviews and meta-analyses published in English were considered, owing to the lack of resources for reliable translation of non-English full texts. We included review articles that evaluated dietary intake of SFA (not biomarkers of fat intake such as plasma or tissue concentrations) using dose–response, high compared with low intakes, or replacement models. For example, we included systematic reviews and meta-analyses reporting on intake of higher compared with lower amounts of SFA in the diet; systematic reviews and meta-analyses reporting on replacing a certain percentage of total fat or calories from SFA with other nutrients; and studies examining high-fat compared with low-fat versions of the same food, provided comparisons were based solely on differences in fat composition, with total consumption of the food being the same, despite the different energy content. We acknowledge that these food-based comparisons typically reflect changes not only in SFA intake but also in total fat, total energy, MUFA, PUFA, trans-fatty acids (TFA), and other nutritional characteristics. This is particularly important for foods with a complex matrix, such as dairy, because high compared with low levels of consumption of those foods are accompanied not only by differences in SFA intake but also by differences in many other nutrients. In this instance, therefore, we included reviews that compared low-fat with high-fat dairy at the same total dairy consumption. For the same reason, we excluded reviews evaluating the cardiovascular effects of whole diets with low SFA content, such as the Mediterranean diet, vegan and vegetarian diets, and the Dietary Approaches to Stop Hypertension diet, as these contrasts are not specific to SFA. Articles examining the association between CVD and other classes of fatty acids, namely PUFA, MUFA, or TFA, but not including SFA, were also not considered.

### Search strategy and study selection

A systematic literature search was conducted across 4 databases: PubMed, Scopus, Web of Science, and Cochrane. We used an inclusive search string that was applied to all field levels and used words and phrasing related to CVD and mortality, SFA consumption, and type of article. The search string was adapted for each database with adjustments to Boolean operators, truncations, and syntax (Supplemental material). The multiplatform search was conducted on 7 March, 2025.

### Article screening

Retrieved articles were imported into an online screening tool and reference management software (www.rayyan.ai) [18]. Removal of duplicates was carried out by 1 team member, prioritizing publication in PubMed or the newest study version available. The systematic reviews and meta-analyses were then screened for inclusion or exclusion at the title and abstract levels independently by 3 team members, and discrepancies were resolved by discussion. Full-text article screening according to the predefined inclusion and exclusion criteria was conducted independently by 2 team members, and a third resolved discrepancies. Upon mutual agreement, papers not relevant to the research question were discarded. To ensure that we included all relevant systematic reviews and meta-analyses, additional searches were conducted in the references of all identified reviews, but also in some previous umbrella reviews [19], [20], [21], [22], [23], [24], [25], [26], [27], [28] retrieved by our search.

### Data extraction and synthesis

An extraction tool was created in Microsoft Excel. Data extraction from full texts was independently carried out by 2 team members, and a third resolved discrepancies. We extracted information regarding the title, authors, number and type of included primary studies specific on SFA, population (number of participants, age, sex), exposures and comparators (type of comparison, level and frequency of intake, dose-dependent or replacement model), and key findings (risk ratio or odds ratio). We extracted only the aggregated results from each systematic review and meta-analysis, and not the individual study-level data. On the basis of these results, and for ease of interpretation, we summarized the conclusions from included reviews by classifying them into 1 of 3 categories regarding the “effect” of SFA: harmful, neutral, or beneficial.

### Bias assessment

The Risk of Bias in Systematic Reviews (ROBIS) tool was used to evaluate the risk of bias in included systematic reviews and meta-analyses. The tool is structured into 3 phases: *1*) assessing relevance (optional); *2*) identifying concerns with the review process; and *3*) judging the overall risk of bias. In the second phase, we evaluated included systematic reviews and meta-analyses across 4 domains where bias might be introduced: *1*) study eligibility criteria, evaluated whether the criteria for including primary studies were clearly defined and appropriate; *2*) identification and selection of studies, assessed if the process was systematic and unbiased; *3*) data collection and study appraisal, checked for consistency and thoroughness; and *4*) synthesis and findings, ensured interpretation was supported by the evidence. For this review, we focused on the second and third phases, omitting the optional first phase. In accordance with the ROBIS guidance, each domain was classified as having low, high, or unclear risk of bias. If “unclear” was assigned, the information reported was insufficient to allow for a confident judgment on the bias concern. Risk-of-bias assessment was performed at the level of the included systematic reviews and meta-analyses rather than at the level of individual primary studies. The risk of bias was evaluated independently by 2 team members, and disagreements were resolved by a third one.

## Results

Across the 4 databases, a total of 4867 records were retrieved (Figure 1). Of those, 3083 records were duplicates; a total of 1582 records were then deleted, as some records were retrieved 3 or 4 times. The remaining 3285 unique records were screened by title and abstract, of which 3239 were excluded because they did not meet the inclusion criteria. The full text of 46 records was assessed, and 22 were excluded due to publication type (*n* = 10), incorrect exposure (*n* = 8), language other than English (*n* = 3), and outcome mismatch (*n* = 1). An additional 7 eligible studies were identified using other methods: 5 were identified through reference searching of previous umbrella reviews [9], [29], [30], [31], [32], 1 that appeared after the search was conducted [33], and another that was not indexed in any of the databases [34]. This resulted in a total of 31 articles for data extraction and synthesis.

**FIGURE 1:**
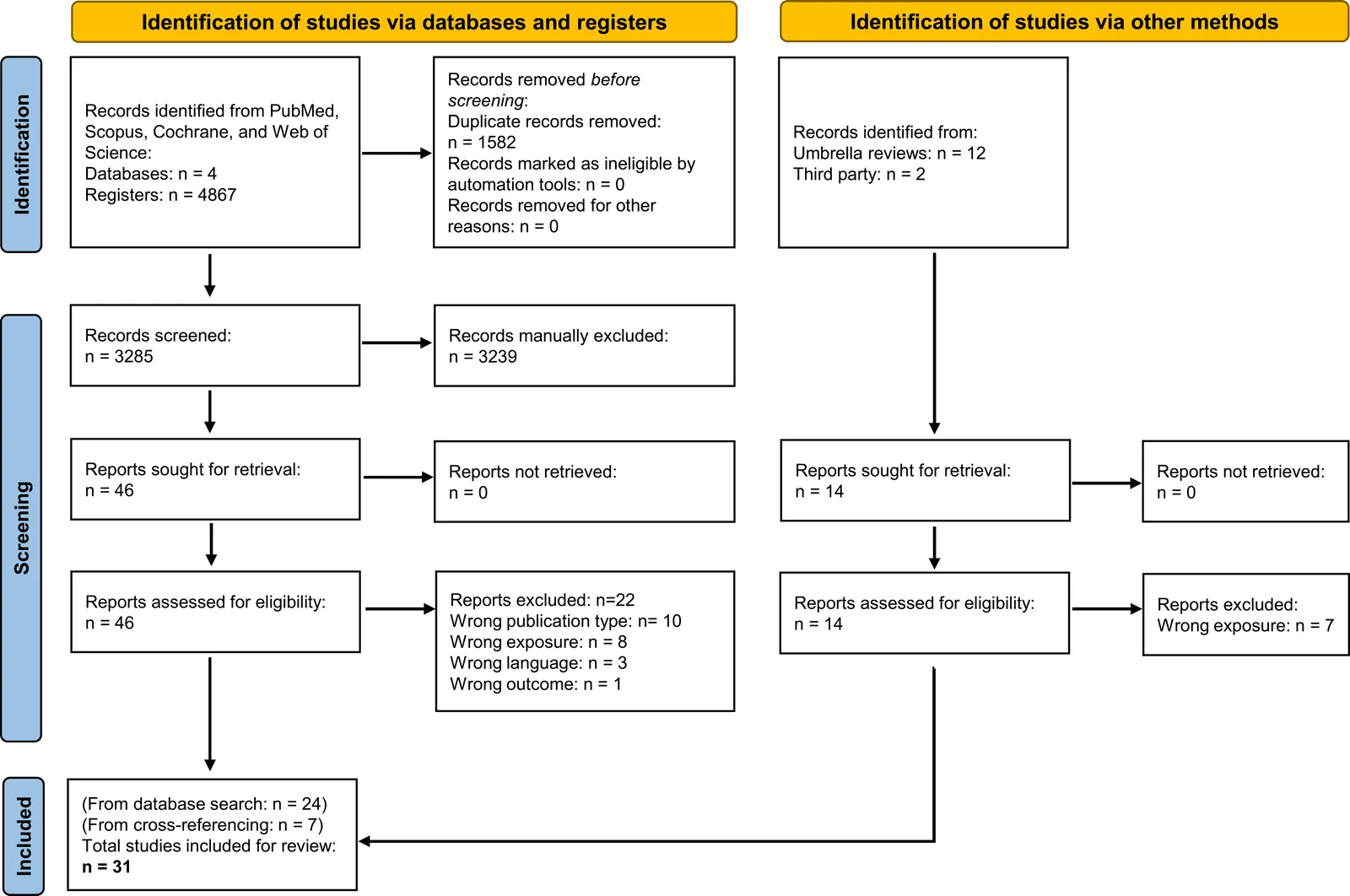
PRISMA flow diagram.

The data extracted are summarized in Table 1 [9], [11], [29], [30], [31], [34], [35], [36], [37], [38], [39], [40], [41], [42], [43], [44], [45], [46], [47], [48], [49], [50], [51] for reviews of observational studies and Table 2 [33], [36], [52], [53], [54], [55], [56], [57] for reviews of RCTs. Our narrative review included a total of 32 analyses from 31 publications: 4 systematic reviews and 28 systematic reviews with meta-analysis. The systematic reviews and meta-analyses identified in the literature search were published between 2001 and 2025, and all reported on the consumption of total SFA from the diet or from specific food groups. Although several primary studies evaluated individual SFA, only a limited number of meta-analyses have synthesized evidence separately for specific SFA [34], [35], [39], with most reviews focusing primarily on total SFA intake [33], [36], [37], [38], [40], [41], [42], [43], [44]. Of the 31 systematic reviews and meta-analyses, 23 reported on data from observational studies [9], [11], [29], [30], [31], [34], [35], [36], [37], [38], [39], [40], [41], [42], [43], [44], [45], [46], [47], [48], [49], [50], [51] and 9 reported on data from RCTs [32], [33], [36], [51], [52], [53], [54], [55], [56], [57]. Attrition rates in the primary studies were typically not reported in the meta-analyses.

**TABLE 1:** Characteristics of included reviews of observational studies and their results

Review	Design of review and included primary studies	Duration (y)	Model of SFA intake assessment	Outcomes and key findings	Takeaway conclusion for SFA intake	Risk of bias (ROBIS)
Adegbola et al. 2022 [45]	• SR • 7 OBS • *n* = 21,280	• 4–10	• Dose–response	• Odds ratio for PAD incidence 5 out of 7 indicated increased risk with higher SFA consumption.	Harmful	Low
Cheng et al. 2016 [37]	• SRMA • 15 OBS cohort • *n* = 476,569	• 7.6–23	• High vs. low	• Stroke incidence, RR = 0.89 (0.82–0.96) • Ischemic stroke, RR = 0.90 (0.82–0.99) • Hemorrhagic stroke, RR = 0.76 (0.62–0.92) • Stroke mortality, RR = 0.75 (0.59–0.94)	Beneficial	Low
Chowdhury et al. 2014 [46]	• SRMA • 32 OBS cohort • *n* = 512,420	• 5–23	• Dose–response	• CAD incidence, RR = 1.03 (0.98–1.07)	Neutral	Low
de Souza et al. 2015 [39]	• SRMA • 41 OBS cohort • All-cause mortality: 7 studies, *n* = 99,906 • CAD mortality: 15 studies, *n* = 101,712 • CVD mortality: 5 studies, *n* = 90,501 • Total CAD: 17 studies, *n* = 267,416 • Ischemic stroke: 15 studies, *n* = 339,090	• 3–32	• Dose–response	Most adjusted estimates: • All-cause mortality, RR = 0.99 (0.91–1.09) • CAD mortality, RR = 1.15 (0.97–1.36) • CVD mortality, RR = 0.97 (0.84–1.12) • CAD incidence, RR = 1.06 (0.95–1.17) • Ischemic stroke incidence, RR = 1.02 (0.90–1.15)	Neutral	Uncertain
Harcombe et al. 2017 [47]	• SR • 6 OBS cohort (5 primary and 1 secondary prevention) • *n* = 31,445	• 4–20 (mean 7.5)	• Dose–response	All-cause and CAD mortality: • Only 1 study reported a direct association between SFA intake and CAD mortality. The majority found no significant associations.	Neutral	Uncertain
Harcombe et al. 2017 [38]	• SRMA • 7 OBS cohort • *n* = 89,801	• 6–20 (mean 11.9)	• Dose–response	• CAD mortality, RR = 1.1 (0.94–1.30)	Neutral	Low
Jakobsen et al. 2009 [48]	• SRMA • 11 OBS cohort • *n* = 344,696	• 4–10	• Replacement	CAD incidence, 5% energy replacement with • PUFA, HR = 0.87 (0.77–0.97) • MUFA, HR = 1.19 (1.00–1.42) • Carbohydrate, HR = 1.07 (1.01–1.14) CAD mortality, 5% energy replacement with • PUFA, HR = 0.74 (0.61–0.89) • MUFA, HR = 1.01 (0.73–1.41) • Carbohydrate, HR = 0.96 (0.82–1.13)	Mixed: harmful (vs. PUFA on incidence and mortality); neutral or beneficial (vs. MUFA or carbohydrate on incidence and mortality)	Low
Jakobsen et al. 2021 [30]	• SRMA • 2 OBS cohort • *n* = 92,097	• 13.4–15.2	• High-fat vs. low-fat dairy intake	Ischemic stroke incidence: • Milk: RR: 0.98 (0.91–1.05) • Yogurt: RR: 1.57 (0.77–3.21)	Neutral	Low
Jayedi et al. 2024 [35]	• SRMA • 26 OBS cohort • *n* = 687,920	• 4.8–30	• Dose–response	• CAD incidence, RR = 1.03 (0.99–1.08)	Neutral	Low
Kang et al. 2020 [42]	• SRMA High vs. low: • 14 OBS cohort • *n* = 598,435 Dose–response: • 12 OBS cohort • *n* = 462,268	• 7.4–20	• High vs. low, dose–response	Stroke incidence: • High vs. low, RR = 0.87 (0.78–0.96) • Dose–response, RR = 0.94 (0.89–0.98)	Beneficial	Low
Kim et al. 2021 [29]	• SRMA • 12 OBS cohort • *n* = 938,386	• 6–32	• Dose–response	All-cause mortality: • Highest vs. lowest, RR = 1.03 (0.94–1.13) • 5% energy increment, RR= 1.02 (1.00–1.05) CVD mortality: • Highest vs. lowest, RR = 1.02 (0.92–1.12) • 5% energy increment, RR = 1.03 (1.00–1.07)	Mixed: neutral (high vs. low); harmful (5% increment)	Low
Ma et al. 2024 [43]	• SRMA All-cause and CVD mortality: • 29 OBS • *n* =1,283,730 CVD incidence: • 31 OBS • *n* = 1,364,664	• 4.6–30	• Dose–response	• All-cause mortality, RR = 1.05 (0.98–1.13) • CVD mortality, RR = 1.03 (0.98–1.08) • CVD incidence, RR = 0.96 (0.92–1.02) • Stroke incidence, RR= 0.92 (0.82–1.02)	Neutral	Uncertain
Mazidi et al. 2020 [40]	• SRMA • 29 OBS cohort • *n* = 1,164,029	• 3.7–32	• Dose–response	• All-cause mortality, HR= 1.04 (0.98–1.11) • CVD mortality, HR = 0.96 (0.84–1.11) • CAD mortality, HR = 1.10 (1.01–1.20); sensitivity analysis HR = 1.03 (0.99–1.08) • Stroke mortality, HR = 1.03 (0.85–1.26)	Neutral	Low
Mente et al. 2009 [9]	• SRMA • 11 OBS cohort • *n* = 160,673	• 2.8–28 (median 11)	• High vs. low	• Composite CAD incidence and mortality, RR = 1.06 (0.96–1.15) • Composite CVD incidence and mortality and total mortality, RR = 1.06 (0.96–1.15)	Neutral	Low
Muto et al. 2018 [41]	• SRMA • 11 OBS cohort • *n* = 423,870	• 7.6–20	• Dose–response	Stroke incidence: • Intracerebral hemorrhage, HR = 0.69 (0.48–1.00) • Ischemic stroke, HR = 0.89 (0.82–0.96)	Beneficial	High
Perna et al. 2022 [11]	• SR • 5 OBS cohort • *n* = 310,618	• 12.2–25.8	• Replacement	CVD incidence, SFA replacement with • PUFA: 2 studies found no association, 1 found mixed results, 1 found lower risk, and 1 found higher risk • MUFA: 2 studies found no association, 1 found lower risk, and 1 found higher risk • Carbohydrates: 2 studies found no association, 1 found lower risk (whole grain), and 1 found higher risk • Proteins: 5 studies found mixed results for plant protein and 2 found higher risk for animal protein	Neutral	High
Reynolds et al. 2022 [34]	• SRMA • 112 OBS cohort • *n* = 3,696,568	• 4–32	• High vs. low, dose–response, replacement	All-cause mortality: • Total SFA, RR = 1.08 (1.00–1.17) • Above vs. below 10%, RR = 1.09 (1.01–1.18) • 5% energy with PUFA, RR = 0.85 (0.75–0.97) • 5% energy with all MUFA, RR = 0.84 (0.75–0.95) • 5% energy with plant MUFA, RR = 0.85 (0.82–0.88) • 5% energy with animal MUFA, RR = 1.00 (0.83–1.20) • 5% energy with carbohydrates, RR = 0.92 (0.86–0.99) • 2% energy with trans-fat, RR = 1.09 (0.97–1.22) CAD incidence (fatal and nonfatal) • Total SFA, RR = 1.04 (0.98–1.12) • Above vs. below 10%, RR = 1.00 (0.87–1.14) • 5% energy with PUFA, RR = 0.89 (0.81–0.98) • 5% energy with all MUFA, RR = 1.00 (0.82–1.21) • 5% energy with plant MUFA, RR = 0.83 (0.69–1.00) • 5% energy with animal MUFA, RR = 1.06 (0.80–1.41) • 5% energy with carbohydrates, RR = 0.98 (0.88–1.09) • 5% energy with proteins, RR = 1.26 (1.06–1.50) • 5% energy with plant proteins, RR = 0.83 (0.61–1.12) • 5% energy with animal proteins, RR = 1.31 (1.14–1.50) • 2% energy with trans-fat, RR = 1.06 (0.89–1.26) CVD incidence (fatal and nonfatal) • Total SFA, RR = 1.07 (0.99–1.17) • Above vs. below 10%, RR = 1.10 (0.99–1.22) • 5% energy with PUFA, RR = 0.90 (0.81–1.00) • 5% energy with MUFA, RR = 0.94 (0.87–1.02) • 5% energy with carbohydrates, RR = 0.98 (0.90–1.07) • 2% energy with trans-fat, RR = 1.07 (0.96–1.20) Ischemic stroke • Total SFA, RR = 0.98 (0.87–1.12) • Above vs. below 10%, RR = 0.91 (0.67–1.24)	Mixed: harmful (dose–response and vs. PUFA, plant MUFA, and carbohydrate on mortality, and vs. PUFA on CAD incidence); beneficial (vs. animal protein on incidence); neutral (most comparisons on CAD and CVD incidence and stroke)	Low
Schwab et al. 2014 [44]	• SR • 6 OBS cohort • *n* = 220,135	• 10–16	• Dose–response, replacement	CVD incidence: • Most studies with dose–response found no association • Studies with replacement found reduced risk with PUFA, and increased risk or no change with carbohydrates	Mixed: harmful (vs. PUFA); neutral or beneficial (vs. carbohydrate)	Low
Schwab et al. 2021 [31]	• SRMA • 5 OBS cohort • *n* = 22,591 with diabetes	• 4.5–11 (mean 9.8)	• Dose–response, replacement	CVD incidence, replacement of SFA with • 2% energy with PUFA, RR = 0.87 (0.77–0.99) • 5% energy with carbohydrates, RR = 0.82 (0.67–1.00) CVD incidence, dose–response: • Extreme quantile SFA intake, RR = 1.13 (0.80–1.59) • Per 10-g SFA increase, RR = 1.35 (0.75– 2.43)	Mixed: harmful (vs. PUFA and carbohydrate); neutral (dose–response)	Low
Siri-Tarino et al. 2010 [49]	• SRMA • 21 OBS cohort • *n* = 347,747	• 5–23	• Dose–response	• CAD incidence, RR = 1.07 (0.96–1.19) • Stroke incidence, RR = 0.81 (0.62–1.05) • CVD incidence, RR = 1.00 (0.89–1.11)	Neutral	Low
Skeaff and Miller 2009 [36]	• SRMA • 28 OBS cohort • *n* = 280,000	• 4–25	• High vs. low, dose–response	High vs. low: • CAD incidence, RR = 0.93 (0.83–1.05) • CAD mortality, RR = 1.14 (0.82–1.60) Dose–response, per 5% energy increment: • CAD incidence, RR =1.03 (0.87–1.22) • CAD mortality, RR = 1.11 (0.75–1.65)	Neutral	High
Wallerer et al. 2024 [50]	• SRMA • 36 OBS cohort • 1,102,268	• 3.7–27.3	• Replacement	All-cause mortality: • Replacement of SFA with PUFA, HR = 0.86 (0.81–0.91) • Replacement of SFA with all MUFA, HR = 0.91 (0.86–0.97) • Replacement of SFA with plant MUFA, HR = 0.85 (0.80–0.90) • Replacement of SFA with animal MUFA, HR = 1.05 (0.99–1.12) • Replacement of trans-fat with SFA, HR =0.87 (0.78–0.97) • Replacement of carbohydrate with SFA, HR = 1.04 (0.99–1.11) • Replacement of protein with SFA, HR = 1.06 (0.96–1.16)	Mixed: harmful (vs. PUFA and plant MUFA); neutral (vs. animal MUFA, carbohydrate and protein); beneficial (vs. trans-fat)	Low
Zhu et al. 2019 [51]	• SRMA • 56 OBS cohort • 1,302,057	• 4–32	• High vs. low, dose–response	CVD incidence: • High vs. low, HR = 0.97 (0.93–1.02) • Per 5% energy increment, HR = 0.99 (0.95–1.04) • Per 5 g/d increment, HR = 0.98 (0.95–1.00)	Neutral	Uncertain

Values in parentheses are 95% CIs.

Abbreviations*:* CAD, coronary artery disease; CVD, cardiovascular disease; OBS, observational study; PAD, peripheral artery disease; SR, systematic review; SRMA, systematic review with meta-analysis.

**TABLE 2:** Characteristics of included reviews of RCTs and their results

Review	Design of review and included primary studies	Duration (y)	Model of SFA intake assessment	Outcomes and key findings	Takeway conclusion for SFA intake	Risk of bias (ROBIS)
Hamley 2017 [52]	• SRMA • 5 “adequately conducted” RCT • *n* = 11,995	• 2–7	• High vs. low (interventions to reduce or replace fat intake)	• CAD incidence, RR = 1.02 (0.84–1.23) • CAD mortality, RR = 1.13 (0.91–1.40) • All-cause mortality, RR = 1.07 (0.90–1.26)	Neutral	High
Harcombe et al. 2015 [53]	• SRMA • 6 RCT • *n* = 6427	• 2–11	• High vs. low (interventions to reduce fat intake)	• CAD mortality, RR = 0.99 (0.78–1.25) • All-cause mortality, RR = 1.00 (0.87–1.15)	Neutral	Low
Hooper et al. 2001 [54]	• SRMA • 11 RCT • *N* = 18,195	• 0.4–9.3 (mean 2.5)	• High vs. low (interventions to reduce fat or cholesterol intake)	• All-cause mortality, RR = 0.98 (0.86–1.12) • CVD mortality, RR = 0.91 (0.77–1.07) • CVD incidence, RR = 0.84 (0.72–0.99)	Mixed: harmful (incidence); neutral (mortality)	Low
Hooper et al. 2020 [55]	• SRMA • 15 RCT • *n* = 56,675	• 2–9 (mean 4.7)	• High vs. low (interventions to reduce fat intake)	• All-cause mortality, RR = 0.96 (0.90–1.03) • CVD incidence, RR = 0.83 (0.70–0.98) • CVD mortality, RR = 0.95 (0.80–1.12) • CAD incidence, RR = 0.83 (0.68–1.01) • CAD mortality, RR = 0.97 (0.82–1.16)	Mixed: harmful (incidence); neutral (mortality)	Low
Mozaffarian et al. 2010 [56]	• SRMA • 8 RCT • *n* = 13,614	• 1–8	• Replacement (interventions to reduce or replace fat intake)	CAD incidence, 5% energy replacement with PUFA: • RR = 0.90 (0.83–0.97)	Harmful	Low
Ramsden et al. 2016 [32]	• SRMA • 5 RCT • *n* = 10,808	• 2–8	• Replacement	Replacement of SFA with PUFA: • CAD mortality, HR = 1.13 (0.83–1.54) • All-cause mortality, HR = 1.07 (0.90–1.27)	Neutral	Low
Schwingshackl and Hoffmann 2014 [57]	• SRMA • 12 RCT • *n* = 7150	• 1–6	• High vs. low (interventions to reduce or replace fat intake)	• CVD mortality, RR = 0.96 (0.66–1.31) • CVD incidence, RR = 0.85 (0.65–1.34) • MI incidence, RR = 0.76 (0.54–1.09) • All-cause mortality, RR = 0.92 (0.68–1.25)	Neutral	Low
Skeaff and Miller 2009 [36]	• SRMA • 7 RCT • *n* = 6694	• 2–8.1	• High vs. low (interventions to reduce or replace fat intake)	Reducing total fat: • CAD mortality, RR = 1.00 (0.80–1.24) • CAD incidence, RR = 0.93 (0.84–1.04) • All-cause mortality, RR = 0.98 (0.90–1.06) Replacement of SFA with PUFA: • CAD mortality, RR = 0.84 (0.62–1.12) • CAD incidence, RR = 0.83 (0.69–1.00) • All-cause mortality, RR = 0.88 (0.76–1.02)	Neutral	High
Yamada et al. 2025 [33]	• SRMA • 9 RCT • n = 13,532	• 2–8	• High vs. low (interventions to reduce or replace fat intake)	• CVD mortality, RR = 0.94 (0.75–1.19) • CAD incidence, RR = 0.85 (0.65–1.11) • MI incidence, RR = 0.85 (0.71–1.02) • All-cause mortality, RR = 1.01 (0.89–1.14)	Neutral	Low

Values in parentheses are the 95% CIs.

Abbreviations: CAD, coronary artery disease; CI, confidence interval; CVD, cardiovascular disease; MI, myocardial infarction; RCT, randomized controlled trial; SRMA, systematic review with meta-analysis.

SFA intake in the included primary observational studies and RCTs ranged from <5% to 10% of total energy intake (the recommended range) to >20%–30% [33], [36], [51]. Dietary SFA intake in relation to CVD outcomes was investigated independently in models involving dose–response, high compared with low intake (e.g., quartiles), or per certain increment in g/d or percent daily energy; or relative to other nutrients in models of replacement by PUFA, MUFA, carbohydrates, TFA, or proteins.

In those systematic reviews and meta-analyses that reported the age of the participants of the included primary studies, ages ranged from 30 to 89 y, and the sex distribution varied, with males being generally overrepresented in RCTs, and males and females being approximately equally represented in observational studies. Some of the systematic reviews and meta-analyses had only a few SFA-specific primary studies, and data for these were extracted separately from the whole review. The duration of follow-up in the included primary studies varied widely from 0.4 to 32 y, largely because of different study designs (observational: 2.8–32 y; RCT: 0.4–9.3 y). According to the ROBIS assessment, the risk of bias in systematic reviews and meta-analyses was low in 23 (71.9%), uncertain in 4 (12.5%), and high in 5 (15.6%). Reviews at high or uncertain risk of bias predominantly reported neutral findings (8 of 9 analyses); the only exception was 1 high-risk review reporting a beneficial association with stroke outcomes [41]. Thus, the predominance of neutral or mixed findings was also evident among the methodologically stronger reviews and was not solely attributable to reviews with a higher risk of bias.

Multiple hard endpoints were assessed, and some systematic reviews and meta-analyses reported on >1 (i.e., all-cause mortality, CAD and stroke incidence, and CAD and stroke mortality). In the 32 analyses, there were 34 assessments of disease incidence (13 CAD, 10 CVD, 8 stroke, 2 myocardial infarction, and 1 peripheral artery disease), 39 assessments of mortality (16 all-cause, 13 CAD, 8 CVD, and 2 stroke), and 2 assessments of composite cardiac endpoints [9], [34] (i.e., combining CAD or CVD events and death).

Most reviews, regardless of study design, reported no significant relationship between intake of SFA and CVD outcomes (i.e., neutral). Two reviews found a harmful effect, one for CAD incidence based on RCTs [56] and the other on peripheral artery disease incidence based on observational studies [45], whereas 3 reviews found a beneficial effect (all on stroke, and only among those of East Asian descent) [37], [41], [42].

Among the observational reviews reporting mixed findings, nutrient replacement models generally found more favorable cardiovascular outcomes when SFA was replaced with PUFA [31], [34], [44], [48], [50]. Overall, only 1 systematic review without a meta-analysis, with a high risk of bias, failed to observe a beneficial effect of replacing dietary SFA with PUFA [11]. Substitution of SFA by MUFA, carbohydrates, proteins, and TFA yielded variable results [11], [31], [34], [44], [48], [50]. There was a beneficial effect when substituting SFA with plant, but not animal, MUFA [34], [50], and a harmful effect when substituting SFA with animal, but not plant, protein [11], [34], [50]. The 2 analyses of RCTs with mixed results were published by the same lead author ∼20 y apart and both reported a harmful effect of SFA on CVD incidence but not on mortality [54], [55].

## Discussion

This narrative review synthesized evidence from 31 systematic reviews and meta-analyses published between 2001 and 2025, encompassing both observational studies and RCTs on dietary SFA intake and CVD outcomes. Despite decades of research and ongoing debate, our findings indicate that the relationship between SFA consumption and CVD risk remains complex and, in many respects, inconclusive.

Across 32 analyses included in the 31 publications, the majority reported no significant association between SFA intake and CVD outcomes, regardless of study design. This neutrality was observed in both observational studies and RCTs, suggesting that high compared with low intake of SFA, or incremental changes in SFA consumption, may not independently predict CVD risk. However, these findings should be interpreted in light of the methodological quality of the reviews included, which was variable across publications and may have influenced the consistency and strength of the reported associations. A minority of reviews identified either harmful or beneficial effects. Two reviews reported adverse outcomes, one linking SFA intake to increased CAD incidence in RCTs and another associating it with peripheral artery disease in observational studies. Conversely, 3 reviews found protective effects, all related to stroke incidence among East Asian populations, highlighting potential ethnic or regional differences in dietary patterns and cardiometabolic responses.

Mixed results were observed primarily in studies employing nutrient substitution models. Notably, 5 observational analyses consistently demonstrated that replacing SFA with PUFA reduced CVD risk [31], [34], [44], [48], [50], supporting the dietary recommendations favoring PUFA over SFA. Substitution effects involving MUFA, carbohydrates, proteins, and TFA were less consistent. For example, substitutions with plant-derived MUFA appeared beneficial, those with animal-derived MUFA and plant-derived protein were associated with neutral outcomes, and those with animal-derived protein appeared harmful. Given that animal foods rich in protein are typically also rich in SFA, translating this relationship into actionable dietary guidelines remains challenging. These findings underscore the importance of considering the nutrients replacing SFA when evaluating the health impact of SFA reduction. What is more, given the nature of the replacement model comparison, the finding that SFA substitution by PUFA is beneficial for heart health could imply either that SFA consumption is inherently harmful or that PUFA consumption is inherently beneficial and not necessarily only when replacing SFA (i.e., also when replacing other dietary nutrients) [50]. And, like SFA, the effect of PUFA on CVD risk may also depend on the food source and matrix [58].

The predominance of neutral findings raises critical questions about the role of SFA in CVD etiology. Several factors may explain this pattern. First, SFA in the diet often coexists with other nutrients in complex food matrices, making it difficult to isolate its independent effects. Thus, discouraging the consumption of certain foods, such as full-fat dairy or grass-fed meats, solely on the basis of their high SFA content may not be appropriate, as these foods contain a complex matrix of multiple other nutrients known to promote health [59]. Second, differences in genetics, gut microbiota composition, physical activity habits, metabolic flexibility, and other factors can influence how SFAs are processed and utilized, potentially attenuating or augmenting their metabolic effects [60], [61]. These principal drivers of interindividual variability in the response to diet are largely understudied in the context of SFA. Third, heterogeneity in study designs and analytical models further contributes to variability in results. Observational studies, although valuable for long-term exposure assessment, are susceptible to residual confounding and measurement error. In addition, reliance on observational data precludes the ability to infer causality or its absence, something particularly important when updating dietary guidelines for the public. Conversely, RCTs offer stronger causal inference, although they are typically short in duration and may not capture the cumulative effects of dietary patterns over decades; they, too, are associated with difficulties in implementation and interpretation. Of the 9 previous systematic reviews that included only RCTs, 6 reported no effect of higher SFA intake on CVD outcomes, 3 reported harm on disease incidence [54], [55], [56], and none reported harm on mortality, whether from CVD or all causes. These reviews, too, have limitations. For instance, in some primary studies reviewed and eventually excluded from the analysis by Hamley [52], only participants in the experimental groups (increased PUFA intake) were instructed to avoid trans-fat, potentially making it difficult to disambiguate results seen in the SFA groups, because substitution of TFA by SFA may be associated with reduced CVD risk [50], albeit not always [34]. Another review by Harcombe et al. [53] included primary studies that implemented different dietary modifications, such as varying PUFA-to-SFA ratios and total fat intakes. Likewise, some primary clinical trials included in the review by Mozaffarian et al. [56] were not truly RCTs but instead nonrandomized trials [33]. In the 2 reviews by Hooper et al. [54], [55], published 20 y apart, a protective effect of reduced SFA intake on CVD incidence was found only when SFA was replaced by PUFA (not by MUFA, carbohydrates, or protein) and only among trials lasting between 2 and 4 y, but not among longer trials (>4 y) [55]. Remarkably, it is important to recognize that the reported benefit was observed for a broad composite endpoint (“combined cardiovascular events,” that include events that may be unrelated to atherosclerotic disease or rarely uniformly defined across trials such as peripheral vascular events), rather than the conventional 3-point or 4-point major adverse cardiovascular event endpoints commonly used in cardiovascular outcome trials. Consequently, the reported reduction in risk may reflect the aggregation of several heterogeneous cardiovascular outcomes, obscuring differences in the magnitude and clinical relevance of effects across individual outcome components. In the updated version including 15 RCTs and over 55,000 individuals, only 1 of the 9 prespecified endpoints turned out to be significant, and when the analysis was restricted to the RCTs that managed to reduce dietary SFA intake (i.e., excluding the studies that aimed to reduce SFA but failed to do so), this effect was no longer significant [55]. Importantly, the metaregression analyses suggested that larger reductions in serum cholesterol, used as a proxy for greater reductions in SFA intake, were associated with greater reductions in cardiovascular events, indicating that the magnitude of dietary change may be an important determinant of effect size. Unfortunately, many of the RCTs in all meta-analyses are limited by their lack of inclusion of adequate numbers of women and healthy individuals, whereas the generally short duration of follow-up, from a few months to less than a decade, allows limited time for putative differences in underlying pathophysiological mechanisms (if any) to manifest and cause symptomatic CVD.

Observational evidence supporting PUFA substitution is consistent with metabolic studies showing PUFA’s favorable effects on intermediate cardiovascular markers, such as lipid profiles and vascular function. However, the link between these biomarkers and hard CVD outcomes remains less definitive. For instance, LDL cholesterol concentration rises with higher dietary SFA [62] and falls when total fat intake is reduced or when SFA is replaced by PUFA. Lowering of LDL cholesterol is considered one of the key mediators of reduced CVD risk [63], but on several occasions, modest diet-induced reductions in LDL cholesterol concentrations do not directly translate into lower CVD risk [32], [52]. In fact, it has been repeatedly demonstrated that changes in classical CVD markers do not always coincide with changes in hard CVD endpoints [64], [65], [66], so relying on LDL cholesterol levels alone to recommend a cap on SFA intake seems inadequate. Beyond their effects on circulating lipoproteins, several additional biological mechanisms have been proposed to explain the relationship between SFA and CVD [67]. Importantly, these mechanisms appear to differ across individual SFA rather than applying uniformly to the entire class. Experimental and mechanistic evidence suggests that specific SFA, particularly palmitic acid (16:0), may influence inflammatory signaling, ceramide synthesis, membrane composition and fluidity, endothelial function, and other metabolic pathways implicated in CVD, whereas other SFA appear to have neutral or even favorable physiological effects [67]. These observations further support the concept that SFA should not be considered as a homogeneous group and reinforce the importance of considering both individual fatty acids and the food matrix in which they are consumed when interpreting CVD risk. Comprehensive discussions of these mechanisms have recently been published elsewhere [67], [68], [69].

Many factors can potentially affect the metabolic effects of dietary SFA, including genetic predisposition, age, sex, and adiposity status [70], but whether and how these factors interact with SFA intake to influence CVD risk is not known. Moreover, the inconsistent findings for MUFA, carbohydrates, and proteins suggest that not all replacements confer equal benefit, and some may even exacerbate risk depending on the source and quality (e.g., refined carbohydrates compared with whole grains, and animal protein compared with plant protein). These nuances highlight the limitations of focusing solely on single nutrients rather than overall dietary patterns. Although some dietary guidelines primarily emphasize replacing SFA with unsaturated fats [71], [72], others support that SFA intake should be as low as possible [13], [73], [74], [75]. Overall, our synthesis suggests that current dietary recommendations are most strongly supported when they emphasize replacement of SFA with PUFA rather than indiscriminate reduction of SFA intake per se. Given that most dose–response systematic reviews and meta-analyses did not identify a consistent association between SFA intake and CVD outcomes, the extent to which a universal quantitative upper limit for SFA intake is supported by the available evidence may reasonably be questioned. By contrast, the most consistent evidence of cardiovascular benefit emerged from replacement analyses, suggesting that the health effects of reducing SFA intake are determined more by what replaces SFA and the resulting overall dietary pattern than by the reduction in SFA intake itself. This is particularly important as consumers are generally not aware of the different types of fat in the diet, their health effects, and the foods containing them [76]. Historically, the simple advice to reduce dietary SFA has led to a decline in total fat consumption and its replacement by carbohydrate [77].

Nutrients are rarely consumed in isolated form, with the notable exception of dietary supplements; in habitual diets, they are typically ingested as components of whole foods. Accordingly, changes in macronutrient composition inherently reflect shifts in specific foods and broader dietary patterns rather than simple exchanges of individual nutrients. Consequently, focusing exclusively on nutrient substitution obscures the heterogeneity of food sources contributing SFA to the diet and fails to capture the complex biological interactions occurring within food matrices that influence digestion, metabolism, and CVD risk. This conceptual limitation has prompted a growing emphasis on food-based substitution analyses, which more accurately reflect habitual eating behaviors and allow evaluation of how replacing SFA-rich foods with alternative food categories influences cardiovascular outcomes [78]. Recent food-based substitution analyses synthesized in the systematic review by Gardner et al. [78] provide more specific evidence on how replacing SFA-rich foods affects CVD risk. Moderate-quality evidence indicates that substituting processed or unprocessed red meat with plant-based protein sources is associated with a lower risk of CVD morbidity. Similarly, replacing red meat with whole grains or vegetables consistently demonstrates reduced CVD risk, supporting the cardioprotective effect of higher-quality carbohydrate sources when displacing SFA-rich animal foods. Instead, the substitution of red or white meat with fish or seafood, or the replacement of red or processed red meat with other animal protein sources (fish, white meat, eggs) is not consistently associated with lower cardiovascular morbidity.

This review’s strengths include its comprehensive scope, inclusion of both observational and experimental evidence, and systematic assessment of bias. Nonetheless, several limitations warrant consideration. Attrition rates in primary studies were rarely reported, and our simple approach to classifying each review’s conclusions as beneficial, neutral, or harmful in relation to SFA intake does not adequately capture heterogeneity in exposure definitions, outcome measures, and statistical models, which undoubtedly complicates direct comparisons across reviews. A key limitation is that we did not quantify overlap among primary studies included across the systematic reviews and meta-analyses, as such an analysis was beyond the scope of the present narrative synthesis. Consequently, the number of included reviews should not be interpreted as representing the same number of independent evidence streams, and our synthesis should be interpreted qualitatively rather than as a count of independent bodies of evidence. A further limitation is the exclusion of non-English publications, which may have introduced language bias and limited the comprehensiveness of the evidence base. Professional translation and linguistic verification were not available, whereas automated translation alone was not considered sufficiently reliable to ensure accurate full-text eligibility assessment and data extraction. We included review articles that evaluated dietary intake of SFA (not biomarkers of fat intake such as plasma or tissue concentrations) using dose–response, high compared with low intake, or replacement models. We included systematic reviews and meta-analyses examining higher-fat compared with lower-fat versions of the same food, implying a reduction that typically reflects an overall reduction in total fat, including SFA, rather than selective modification of individual fatty acid subclasses. However, we acknowledge that differences in fat content may also involve changes in MUFA, PUFA, TFA, total energy content, and other nutritional characteristics.

Our findings suggest that blanket recommendations to reduce SFA intake may oversimplify a complex relationship. Although replacing SFA with PUFA appears beneficial, the health impact of other substitutions remains uncertain and may depend on food sources and dietary context. These effects appear to largely depend on other bioactive substances in food and its matrix.

Importantly, confidence in these conclusions should be considered alongside the methodological quality of the underlying evidence base, as not all included systematic reviews and meta-analyses were judged to have the same risk of bias. Nevertheless, the predominance of neutral or mixed findings persisted when attention was restricted to reviews at low risk of bias, suggesting that the overall uncertainty cannot be explained solely by methodological limitations of the included reviews. Conversely, isolated beneficial or harmful findings, particularly those derived from reviews at high or uncertain risk of bias, should be interpreted more cautiously. Evidence concerning the replacement of SFA with PUFA was comparatively more consistent and was supported by several reviews judged to be at low risk of bias. This could help explain why foods such as yogurt, cheese, and dark chocolate may have beneficial effects on CVD despite their high SFA content [12], [79], [80], [81]. Future research should prioritize high-quality RCTs with longer follow-up, standardized exposure assessment, and exploration of substitution effects in diverse populations. In addition, future studies should more precisely characterize the magnitude of achieved reductions in SFA intake, determine whether specific intake thresholds are required to influence hard cardiovascular outcomes, and distinguish the effects of replacing SFA with different PUFA subclasses, as well as with different carbohydrate and protein sources. Such studies would help clarify the conditions under which reducing SFA intake is most likely to confer cardiovascular benefit. Additionally, greater attention to food-based approaches (dietary patterns), rather than nutrient-centric models, may yield more actionable guidance for public health.

In conclusion, this summary of the available systematic reviews and meta-analyses suggests that the relationship between dietary SFA intake and CVD is more complex than can be captured by a single quantitative target. Overall, the evidence remains heterogeneous, with substantial uncertainty surrounding the independent effects of SFA intake on cardiovascular outcomes. The most consistent finding from observational data is that replacing SFA specifically with PUFA reduces risk, emphasizing consumption of plant-based foods and oils, nuts and seeds, and fatty fish. Overall, the importance of the dietary context in which SFA is consumed, including both food sources and replacement nutrients, emerges. Major health organizations broadly agree on limiting SFA intake to ≤10% of total daily energy and promoting dietary patterns low in SFA. However, recommendations differ in the use of more restrictive targets for specific populations, the emphasis placed on particular replacement nutrients, and the translation of nutrient-based targets into food-based and pattern guidance. Overall, the available evidence supports continued emphasis on balanced dietary patterns, replacement nutrient quality, and food sources, rather than focusing narrowly on a single nutrient target in isolation.

## Author contributions

The authors’ responsibilities were as follows – FM, AA: contributed to conceptualization; FM, JMM, AA: contributed to investigation, data curation and analysis; NT: contributed to funding acquisition; FM, JMM: contributed to writing – original draft; MA, CA, AA, JTB, JCK, RM, RHE, JOH, RMK: contributed to writing—review and editing; FM: contributed to project administration and supervision; and all authors: read and approved the final manuscript.

## Declaration of generative AI and AI-assisted technologies in the writing process

During the preparation of this work, the authors used ChatGPT (OpenAI) to improve the clarity of the writing and refine the use of English.

## Funding

This research was funded by a contribution from the Italian Ministry of Health (current research, RC 2026). The work reported in this manuscript was partially supported by The Nutrition Coalition.

## Conflict of interest

FM is an Associate Editor for *Clinical Nutrition* but played no role in the Journal’s evaluation of the manuscript. NT receives royalty payments for a book on saturated fats and is the Director of the Nutrition Coalition. The authors report no conflicts of interest relevant to the content of this manuscript.
