# Supplementary material for: Revisiting the Evidence Underlying Dietary Recommendations on Saturated Fats and Cardiovascular Disease: A Narrative Synthesis of Systematic Reviews and Meta-Analyses
Source: Adv Nutr. 2026 Oct 7;17(11):100746. doi: 10.1016/j.advnut.2026.100746 (PMC13737732; doi:10.1016/j.advnut.2026.100746)
Supplement: Supplementary file 1 [file mmc1.docx]

**Revisiting the Evidence Underlying Dietary Recommendations on Saturated Fats and Cardiovascular Disease: a Narrative Synthesis of Systematic Reviews and Meta-Analyses**

*Julie Marvel Mansfeldt et al.*

**Supplementary material**

**S1: Search strings used in the four databases**

**PUBMED**

(((cardiac[tw] OR cardiovascular[tw] OR cardiometabolic[tw] OR heart[tw] OR mortality[tw] OR atherosclerosis[tw] OR arteriosclerosis[tw] OR stroke[tw] OR "myocardial infarction"[tw] OR "ischemic heart disease"[tw] OR cvd[tw] OR chd[tw]) AND (disease*[tw] OR event*[tw] OR disorder*[tw] OR complication*[tw] OR mortality[tw] OR prevention[tw] OR incidence[tw] OR occurrence[tw] OR recurrence[tw] OR outcome*[tw])) AND ("saturated fat*"[tw] OR "fatty acid*"[tw] OR sfa[tw] OR "high fat diet*"[tw] OR "animal fat*"[tw] OR "dietary fat*"[tw] OR "full fat"[tw] OR "fried food*"[tw] OR butter[tw] OR oil[tw] OR margarine[tw] OR cream[tw] OR cheese[tw] OR dairy[tw] OR meat[tw] OR lauric[tw] OR laurate[tw] OR myristic[tw] OR myristate[tw] OR palmitic[tw] OR palmitate[tw] OR stearic[tw] OR stearate[tw] OR "reduced fat"[tw])) AND ("systematic review*" OR "meta-analysis" OR "meta-analyses" OR umbrella)

**SCOPUS**

((TITLE-ABS-KEY(cardiac OR cardiovascular OR cardiometabolic OR heart OR mortality OR atherosclerosis OR arteriosclerosis OR stroke OR {myocardial infarction} OR {ischemic heart disease} OR cvd OR chd) AND TITLE-ABS-KEY(disease* OR event* OR disorder* OR complication* OR mortality OR prevention OR incidence OR occurrence OR recurrence OR outcome*)) AND TITLE-ABS-KEY("saturated fat*" OR "fatty acid*" OR sfa OR "high fat diet*" OR "animal fat*" OR "dietary fat*" OR {full fat} OR "fried food*" OR butter OR oil OR margarine OR cream OR cheese OR dairy OR meat OR lauric OR laurate OR myristic OR myristate OR palmitic OR palmitate OR stearic OR stearate OR {reduced fat})) AND TITLE-ABS-KEY("systematic review*" OR "meta-analysis" OR "meta-analyses" OR umbrella)

**WEB OF SCIENCE – CORE**

((TS=(cardiac OR cardiovascular OR cardiometabolic OR heart OR mortality OR atherosclerosis OR arteriosclerosis OR stroke OR “myocardial infarction” OR “ischemic heart disease” OR cvd OR chd) AND TS=(disease* OR event* OR disorder* OR complication* OR mortality OR prevention OR incidence OR occurrence OR recurrence OR outcome*)) AND TS=("saturated fat*" OR "fatty acid*" OR sfa OR "high fat diet*" OR "animal fat*" OR "dietary fat*" OR “full fat” OR "fried food*" OR butter OR oil OR margarine OR cream OR cheese OR dairy OR meat OR lauric OR laurate OR myristic OR myristate OR palmitic OR palmitate OR stearic OR stearate OR “reduced fat”)) AND TS=("systematic review*" OR "meta-analysis" OR "meta-analyses" OR umbrella)

**COCHRANE (REVIEWS)**

(((cardiac OR cardiovascular OR cardiometabolic OR heart OR mortality OR atherosclerosis OR arteriosclerosis OR stroke OR (myocardial NEXT infarction) OR (ischemic NEXT heart NEXT disease) OR cvd OR chd) AND (disease* OR event* OR disorder* OR complication* OR mortality OR prevention OR incidence OR occurrence OR recurrence OR outcome*)) AND ((saturated NEXT fat*) OR (fatty NEXT acid*) OR sfa OR (high NEXT fat NEXT diet*) OR (animal NEXT fat*) OR (dietary NEXT fat*) OR (full NEXT fat) OR (fried NEXT food*) OR butter OR oil OR margarine OR cream OR cheese OR dairy OR meat OR lauric OR laurate OR myristic OR myristate OR palmitic OR palmitate OR stearic OR stearate OR (reduced NEXT fat))) AND ((systematic NEXT review*) OR (meta NEXT analysis) OR (meta NEXT analyses) OR umbrella)
